# Exosomal miRNA signatures of pancreatic lesions

**DOI:** 10.1186/s12876-020-01287-y

**Published:** 2020-05-06

**Authors:** Caterina Vicentini, Federica Calore, Giovanni Nigita, Paolo Fadda, Michele Simbolo, Nicola Sperandio, Claudio Luchini, Rita T. Lawlor, Carlo Maria Croce, Vincenzo Corbo, Matteo Fassan, Aldo Scarpa

**Affiliations:** 1 ARC-NET Research Centre, University of Verona, Verona, Italy; 2 Department of Cancer Biology and Genetics and Comprehensive Cancer Center, Ohio State University, Columbus, Ohio USA; 3 Department of Diagnostics and Public Health, Section of Anatomical Pathology, University and Hospital Trust of Verona, Verona, Italy; 4 Department of Medicine (DIMED), Surgical Pathology and Cytopathology Unit, University of Padua, Via Aristide Gabelli 61, 35121 Padua, PD Italy

**Keywords:** Pancreatic lesions, Circulating miRNAs, Early biomarkers, Exosomes, NanoString profiling

## Abstract

**Background:**

Pancreatic and peri-pancreatic neoplasms encompass a variety of histotypes characterized by a heterogeneous prognostic impact. miRNAs are considered efficient candidate biomarkers due to their high stability in tissues and body fluids. We applied Nanostring profiling of circulating exosomal miRNAs to distinct pancreatic lesions in order to establish a source for biomarker development.

**Methods:**

A series of 140 plasma samples obtained from patients affected by pancreatic ductal adenocarcinoma (PDAC, *n* = 58), pancreatic neuroendocrine tumors (PanNET, *n* = 42), intraductal papillary mucinous neoplasms (IPMN, *n* = 20), and ampulla of Vater carcinomas (AVC, *n* = 20) were analyzed. Comprehensive miRNA profiling was performed on plasma-derived exosomes. Relevant miRNAs were validated by qRT-PCR and in situ hybridization (ISH).

**Results:**

Lesion specific miRNAs were identified through multiple disease comparisons. Selected miRNAs were validated in the plasma by qRT-PCR and at tissue level by ISH. We leveraged the presence of clinical subtypes with each disease cohort to identify miRNAs that are differentially enriched in aggressive phenotypes.

**Conclusions:**

This study shows that pancreatic lesions are characterized by specific exosomal-miRNA signatures. We also provide the basis for further explorations in order to better understand the relevance of these signatures in pancreatic neoplasms.

## Background

Pancreatic ductal adenocarcinoma (PDAC) represents approximately the 90% of all pancreatic cancers and is currently the third leading cause of cancer deaths in the United States with a reported 5-year survival rate of 8% [1]. Early symptoms of PDAC are non-specific, therefore more than half patients are diagnosed at a late stage when the tumor is unresectable [2, 3].

The second most common neoplasm of the pancreas is pancreatic neuroendocrine tumor (PanNET), a rare epithelial malignancy arising from pancreatic islet cells [4]**.** At present, surgical resection of PanNET with curative intent remains the most effective therapeutic option, with systemic therapies being largely ineffective for unresectable diseases [5]. Even if PDAC and PanNET represent the two most common pancreatic cancers, they show completely different genetic profiles [6, 7].

Several risk factors associated to PDAC development have been identified including genetic syndromes, smoking, alcohol and chronic pancreatitis (CP) [8, 9]. In particular, CP has been proposed as independent risk factor for pancreatic cancer development [10].

Three main PDAC precursor lesions have been identified: pancreatic intraepithelial neoplasia (PanIN), intraductal papillary mucinous neoplasm (IPMN) and mucinous cystic neoplasm (MCN), whose early detection and management could prevent progression to malignancy [11]. Several studies have focused their attention on the genetic heterogeneity of pancreatic precursor lesions in order to elucidate the timing of specific alterations in pancreatic tumorigenesis especially in the contest of IPMN and PDAC [12–14].

Ampulla of Vater carcinomas (AVC) comprise a percentage ranging from 15 to 30% of all pancreatico-duodenectomies and 10 to 20% of all tumor-related obstructions of the common bile duct [15, 16].

Although AVC show a better prognosis than PDAC, it remains a deadly disease with a mortality rate of 60% [17, 18]. At present, diagnostic imaging is not able to distinguish between periampullary neoplasms such as periampullary PDAC or periampullary carcinoma of the duodenum and AVC [19].

The current therapeutic approaches for treating advanced pancreatic/peri-pancreatic cancers are poorly effective, and the known common biomarkers are inadequate for a reliable risk stratification as well as in the setting of early diagnosis. Therefore, new non-invasive strategies and more precise therapeutic targets are urgently needed to improve patients’ management and outcome. MicroRNAs (miRNAs) are a class of endogenous, small (19 to 25 nucleotides), non-coding RNAs that modulate the expression of at least one third of protein-coding genes [20–24]. Moreover, miRNAs have been detected within body fluids, such as plasma, serum, urine and breast milk. The so-called “circulating microRNAs” can be found either encapsulated in cell-secreted vesicles or vesicle-free and instead be associated to AGO protein-positive ribonucleoprotein (RNP) particles [25]. It has been demonstrated that secreted miRNAs can be taken up by recipient cells and act as biologically active molecules [26, 27].

It has been widely reported in literature the aberrant miRNAs expression in human cancers including PDAC, its precursor lesions, PanNETs and AVCs both on tissue and biofluids [28–32]. Many genomic aberrations have been associated to the differential expression of miRNAs between normal and malignant tissues as chromosomal alterations, DNA point mutations, epigenetic mechanisms and miRNA processing machinery alterations [21].

On these grounds, the aim of this study was to identify circulating miRNAs able in discriminating the different histotypes of pancreatobiliary neoplasms. Furthermore, we also sought to identify miRNAs differentially expressed in the same histotype, based on biological and clinical differences. In particular, we leveraged the presence of metastatic and localized PDAC in our cohort to identify markers of dismal disease. To our knowledge, this is one of the most extensive study cohort of circulating miRNAs profiling among patients with different pancreatobiliary lesions using digital technology.

## Methods

### Pancreatic lesion patients and clinical samples

A series of 155 plasma samples obtained from 58 PDAC, 42 PanNETs, 20 IPMN, 20 AVC (Additional file 1), and 15 CP patients were retrieved from the archives of the ARC-NET biobank of the Verona University Hospital. All experiments were performed in accordance with relevant guidelines and regulations. Clinicopathological features of PanNET, PDAC and AVC patients are reported in Table 1. Regarding IPMN samples, only 6 of 20 displayed associated invasive pancreatic ductal adenocarcinoma. All peripheral fasting blood samples were collected in 10-mL sodium heparin tubes (BD Vacutainer; Becton-Dickinson, Milan, Italy) and processed within 2 h by centrifugation at 2000 g at 4 °C for 10 min. The plasma obtained was then transferred to new tubes and centrifuged at 3000 g at 4 °C for 10 min to remove platelet contamination. Final plasma preparations were carefully collected from the upper portion of the supernatant and stored in aliquots at − 80 °C.

**Table 1:** Clinicopathological characteristics of considered PDAC, PanNET and AVC

Variable	PDAC (*n* = 55)	PanNET (*n* = 42)	AVC (*n* = 19)^b^
Gender			
F	22 (40%)	16 (38%)	10 (53%)
M	33 (60%)	26 (62%)	9 (47%)
Age (years)	64.5 ± 10,5	51.1 ± 13.65	63.4 ± 11.18
	(median 67.0)	(median 49.5)	(median 63)
Primary tumor			
T1	5 (9%)	20 (47%)	3 (15%)
T2	25 (46%)	15 (36%)	6 (30%)
T3	9 (16%)	7 (17%)	9 (45%)
T4	6 (11%)	0 (0%)	0 (0%)
^a^n.a.	10 (18%)	0 (0%)	1 (5%)
Regional lymph nodes			
N0	3 (6%)	22 (52%)	7 (35%)
N1	15 (27%)	12 (29%)	7 (35%)
N2	21 (38%)	0 (0%)	4 (20%)
n.a.	16 (29%)	8 (19%)	1 (5%)
Distant metastasis			
M0	38 (69%)	40 (95%)	18 (90%)
M1	11 (20%)	2 (5%)	1 (10%)
n.a.	6 (11%)	0 (0%)	0 (0%)
Clinical stage			
I	3 (6%)	11 (26%)	6 (30%)
II	15 (27%)	11 (26%)	0 (0%)
III	20 (36%)	10 (24%)	12 (60%)
IV	11 (20%)	2 (5%)	1 (5%)
n.a.	6 (11%)	8 (19%)	0 (0%)
Grade			
G1	0 (0%)	29 (69%)	2 (10%)
G2	25 (45.5%)	13 (31%)	12 (60%)
G3	14 (25.5%)	0 (0%)	4 (20%)
n.a.	16 (29%)	0 (0%)	1 (5%)
Lymphovascular invasion			
absent	1 (2%)	17 (40.5%)	3 (15%)
present	38 (69%)	17 (40.5%)	15 (75%)
n.a.	16 (29%)	8 (19%)	1 (5%)
Perineural invasion			
absent	0 (0%)	22 (52%)	5 (25%)
present	39 (71%)	12 (29%)	13 (65%)
n.a.	16 (29%)	8 (19%)	1 (5%)

^a^n.a (not available data): patients who did not undergo radical surgery; ^b^One out of 20 AVCs is represented by an adenomatous lesion with high-grade dysplasia (this case has not been reported in the table)

### Preparation of exosomal RNA from pancreatic lesion plasma samples

Plasma-derived exosomes were isolated as described in Casadei et al. [33] by using the ExoQuick system (System biosciences) according to the manufacturer’s protocol. The quality and size of particles were determined by Nanosight, qRT-PCR and western blot analyses (Additional file 2). The exosomal pellet was resuspended in 1 ml Trizol (Life Technologies), and total RNA was isolated with Norgen RNA clean-up and concentration kit (Norgen BioTek), following the provided instructions. Exosomal RNA was eluted in 20 μl of H_2_O; the average RNA yield per group per 1 ml of plasma was: for the CP group ~ 4 ng/ml, for IPMN ~ 6 nm/ml, for PDAC ~ 6.3 ng/ml, for AVC ~ 20 ng/ml, for PanNET ~ 4.7 ng/ml. Nanodrop 1000 spectrophotometer (Thermo Scientific) was used to exclude within our samples the presence of 230 nm peaks, due to organics compounds or chaotropic salts, which could represent a potential issue for the ligase enzymatic step during NanoString profiling.

### Nanoparticle tracking analysis (NTA)

Concentration and particle size distribution in plasma-derived exosomal samples were determined by NTA using NanoSight NS300 system (Malvern Technologies, Malvern, UK). Nanosight was configured with a 532 nm green laser, a high sensitivity scientific CMOS camera, and samples were loaded by using a syringe. Samples were diluted in particle-free PBS (Sigma) to acceptable concentrations based off of the manufacturer’s recommendation. Sample data files were acquired under continuous flow (flow rate = 25–50) and at room temperature. Samples were recorded for 3 × 60 s successive videos and were captured with a camera level of 12. Data were analyzed using NTA 3.1.5 software with a detection threshold of 3–4.

### NanoString nCounter assay and data analysis

A total of 155 samples were processed with Nanostring nCounter® Human v3 miRNA Expression Assay. NanoString analysis was performed as described in Drusco et al. [34]. Briefly, 3.5 μL of exosomal RNA were annealed with multiplexed DNA tags (miR-tag) and bridges target specifics. Mature miRNAs were then bond to specific miR-tags using a Ligase enzyme and all the tags in excess were removed by enzyme clean-up step. The tagged microRNAs product was diluted 1 to 5 and 5 μL were combined with 20 μL of Reported Probes in hybridization buffer and 5 μL of Capture probes. The overnight hybridization (16 to 20 h) at 65 °C allowed to complex probes sequence specific with targets. Probe excess was removed using two-step magnetic beads-based purification on an automated fluidic handling system (nCounter Prep Station) and target/probe complexes were immobilized on the cartridge for data collection. The nCounter Digital Analyzer collected the data by taking images of immobilized fluorescent reporters in the sample cartridge with a CCD camera through a microscope objective lens. For each cartridge, a high-density scan encompassing 325 fields of view was performed. Images were processed internally into a digital format (RCC files). NanoString raw data were analyzed with nSolver™, a software provided by NanoString Technologies. Negative controls were used to perform background subtraction. Positive controls were used to perform technical normalization to adjust any lane-by-lane variability due to differences in hybridization, purification or binding. After technical normalization, the data were biologically normalized by calculating the geometric mean of the top 100 miRNAs in all samples, as recommended by NanoString. *p*-values were calculated using the LIMMA package (Linear Models for Microarray Data) from the Bioconductor R project. The *p*-values were adjusted for multiple testing using the Benjamini and Hochberg method [35] to control the False Discovery Rate (FDR). For each pairwise analysis, we considered all those significant (*p*-value< 0.05) and expressed (≥20 counts in at least one condition, as 20 counts is approximately the average of the expression of the negative controls in the NanoString panel) deregulated miRNAs for the downstream analysis.

### Quantitative real-time PCR

The expression of an exosomal individual mature miRNA was assessed in triplicate by using the TaqMan Stem-loop miRNA assay, according to the manufacturer’s protocol (ThermoFisher). For retro-trancription, 0.5 ng of RNA were used per reaction in a final volume of 7.5 μl. For qRT-PCR, 1 μl of cDNA was used per reaction. For RNA normalization, ath-miR-159a, osa-miR-414, and cel-miR-248 synthetic oligos (Integrated DNA Technologies) were added to each sample right after the exosomal pellet was resuspended in Trizol. Normalization was performed with the 2^-Δct^ method. Results were analyzed by using a two-tailed Student *t* test.

### Western blot

For the western blot analysis, exosomal proteins were isolated from 500 μl of plasma (the yield of exosomal protein was 9 mg/sample). The exosomal pellet was resuspended in RIPA buffer (Cell Signaling technology), supplemented with phosphatase and protease inhibitors (Roche), and incubated in ice for 20′. Samples were then harvested at 14,000 x g for 10′, and the supernatant collected in a new eppendorf. Protein concentration was determined by using Bradford Assay (Bio-Rad), following the manufacturer’s instructions. 80 μg of exosomal lysate were then loaded on a Criterion Tris-HCl 4–20% pre-cast gel (Bio-Rad), transferred onto a nitrocellulose membrane (Bio-Rad) and probed with anti-Alix (1:1000), anti-TSG101 (1:1000), anti-Calnexin (Sigma) (1:2000), and anti-CD9 (Cell Signaling Technology) (1:1000) primary antibodies, followed by isotype matched, horseradish-peroxidase-conjugated secondary antibodies. Finally, the proteins of interest were detected through chemi-luminescence reaction.

### miRNA in situ hybridization analysis (ISH)

Locked nucleic acid (LNA) probes with complementarity to miR-4454, miR-106a-5p, and miR-17-5p were labelled with 5′-biotin and synthesized using Exiqon (Vedbaek, Denmark). Tissue sections were digested with ISH protease 1 (Ventana Medical Systems, Milan, Italy) and ISH was performed as we previously described [36]. Positive (U6; Exiqon) and negative scrambled LNA probes (Exiqon) were used as controls. Only cytoplasmic miRNA staining was retained for scoring purposes.

## Results

### Digital profiling identifies circulating miRNAs specific to the neoplastic state

**Fig. 1:**
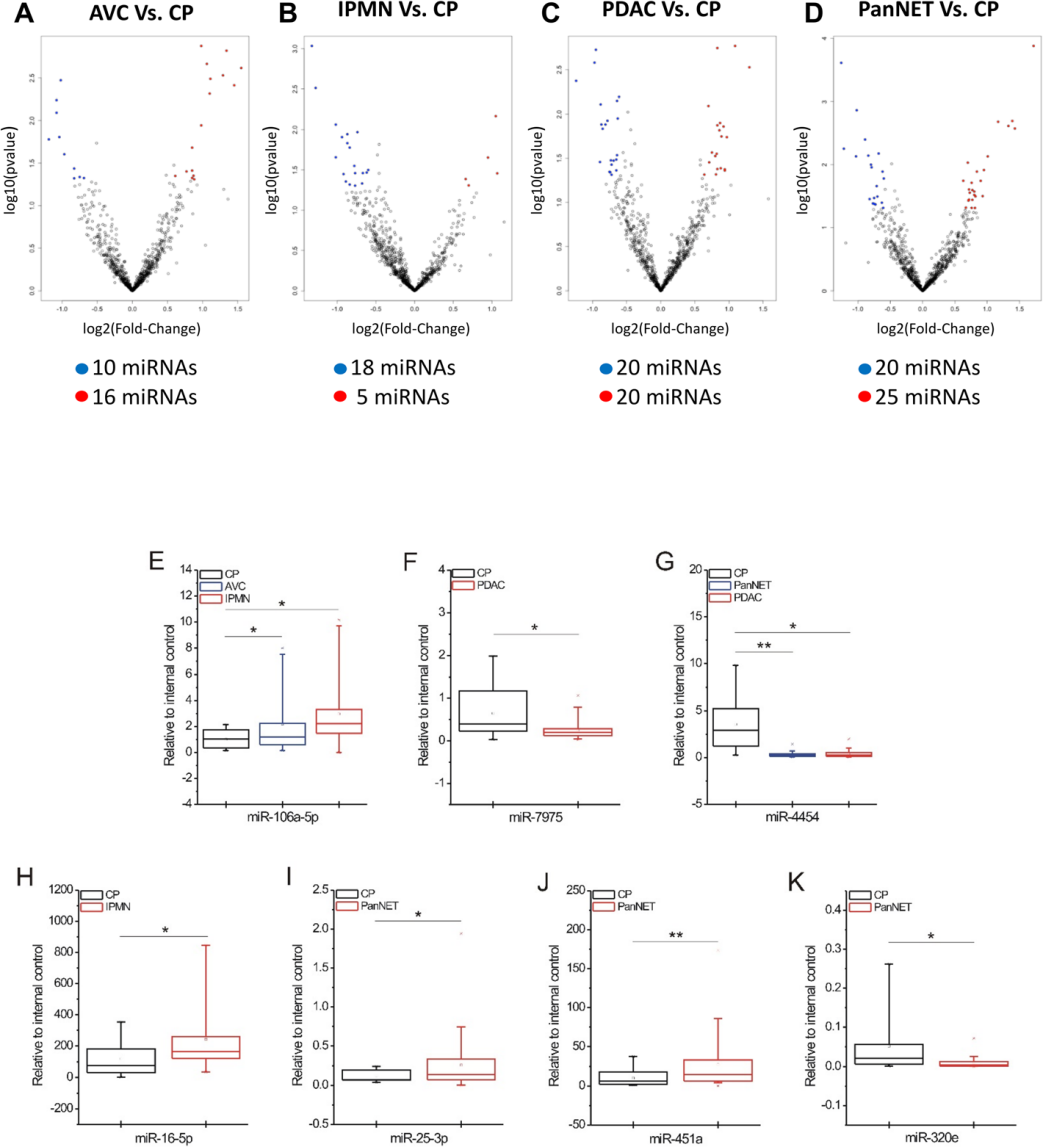
Differential expression of circulating miRNAs in pancreatic lesions compared to chronic pancreatitis. **a-b-c-d** Volcano plots of miRNAs expression showing significant (*P* < 0.05) and deregulated (with a |LinearFC| > 1.5) miRNAs in each comparison. **e-f-g-h-i-j-k** NanoString results were validated by qRT-PCR analysis. The expression levels of the indicated miRNAs in the CP group (here considered as a control group) were compared with the expression of the same miRNAs within the indicated pancreatic cancer groups. The square inside the box plot indicates the mean value, whereas the “x” outside the box indicates the 99-percentile. Student’s *t*-test was performed for statistical analysis. *, 0.01 < *P* ≤ 0.05; **, 0.001 < *P* ≤ 0.01

Comprehensive miRNA profiling was performed in order to identify exosomal miRNAs differently expressed between pancreatic lesions (AVC, IPMN, PDAC and PanNET) and chronic pancreatitis (CP). Overall, we found 26, 23, 40 and 45 deregulated miRNAs between AVC vs CP, IPMN vs CP, PDAC vs CP and PanNET vs CP, respectively (Fig. 1 a-b-c-d). For each comparison, a linear fold change > 1.5 was used as threshold. Next, relevant miRNAs were filtered again considering only those with a number of counts greater than 20 (See Methods section, Table 2 and Additional file 3). In details, we found 5 deregulated miRNAs (3 upregulated and 2 downregulated miRNAs) between AVC and CP; 4 miRNAs between IPMN and CP (3 upregulated and 1 downregulated miRNAs); 9 miRNAs between PDAC and CP (3 upregulated and 6 downregulated miRNAs) and 11 miRNAs between PanNET and CP (6 upregulated and 5 downregulated miRNAs) (Table 2 and Additional file 3).

**Table 2:** Summary of deregulated miRNAs from Nanostring profiling between pancreatic lesions

Type of comparison	Upregulated miRNAs	Downregulated mRNAs
AVC Vs. CP	**miR-106a-5p**; miR-17-5p; miR-520f-3p	miR-4454; miR-7975
IPMN Vs. CP	**miR-106a-5p**; miR-17-5p; **miR-16-5p**	miR-122-5p
PDAC Vs. CP	miR-372-3p; miR-140-3p; miR-644a	miR-1299; miR-146a-5p; miR-148b-3p; miR-130a-3p; **miR-4454**; **miR-7975**
PanNET Vs. CP	**miR-451a**; miR-372-3p; miR-106a-5p; miR-17-5p; **miR-25-3p**; miR-644a	miR-22-3p; miR-1246; miR-4454; **miR-7975**; **miR-320e**
ACV Vs. PDAC	**miR-106a-5p**; **miR-17-5**p; miR-2116-5p; miR-199a-3p; miR-199b-3p; miR-342-3p; miR-520d-5p; miR-527; miR-518a-5p; miR-93-5p; miR-30a-5p; miR-27b-3p; miR-146a-5p; miR-451a; miR-20a-5p; miR-20b-5p; miR-185-5p; miR-520 h; miR-25-3p; miR-1322; miR-19a-3p; miR-302e	–
IPMN Vs. PDAC	miR-4454; miR-7975; miR-2116-5p; miR-1910-5p; **miR-16-5p**; miR-451a; miR-19b-3p; **miR-106a-5p**; **miR-17-5p**; miR-629-5p; miR-376a-3p; miR-20a-5p; miR-20b-5p; miR-26b-5p; miR-93-5p; miR-25-3p; miR-590-5p; miR-30e-5p; **miR-19a-3p**; miR-608; let-7b-5p	–
PanNET Vs. PDAC	miR-451a; miR-26b-5p; miR-25-3p; miR-16-5p	miR-1322; miR-1285-5p; miR-320e
Met. PDAC Vs. Loc. PDAC	miR-106a-5p; miR-17-5p; miR-342-3p; miR-20a-5p; miR-20b-5p; miR-223-3p; miR-16-5p; miR-19b-3p; miR-130a-3p; miR-25-3p; miR-451a	–
PB AVC Vs. INT. AVC	miR-579-3p; miR-422a; miR-1253; miR-3144-3p; miR-1268a; miR-190a-3p	miR-3613-3p; miR-582-5p; miR-1976; miR-885-5p; miR-122-5p
IPMN-C Vs. IPMN	miR-1293; miR-450a-5p; miR-433-5p; miR-324-5p; miR-941; miR-499a-5p; miR-4787-5p; miR-139-3p; miR-516a-3p; miR-516b-3p; miR-665	miR-520f-3p; miR-126-3p

Significant (*P*value< 0.05) and expressed (≥20 counts in at least one condition) deregulated (with a |LinearFC| > 1.5) miRNAs in each comparison. miRNAs validated by qRT-PCR are indicated as bold text

Seven miRNAs were therefore selected for orthogonal validation by qRT-PCR on the same cohort of patients: miR-106-5p, miR-7975, miR-4454, miR-16-5p, miR-25-3p, miR-320e and miR-451a (Fig. 1 e-k and Table 2). In particular, since the NanoString assay is not able to discriminate between miR-106a-5p/miR-17-5p and miR-4454/miR-7975, we decided at first to perform a qRT-PCR analysis for these four miRNAs in order to understand their specific expression contribution within selected sample groups. At first, we found a significant upregulation of miR-106-5p in AVC and IPMN compared to CP (Fig. 1e) while miR-17-5p was not differentially deregulated between the same lesions (data not shown). The expression of both miRNAs was also investigated between PanNET and CP showing no different between groups (data not shown). Than we validated the downregulation of miR-7975 and miR-4454 in PDAC compared to CP while only miR-4454 was found downregulated in the comparison between PanNET and CP (Fig. 1 f-g). The upregulation of miR-16-5p was also validated by qRT-PCR in IPMN groups, while the upregulation of miR-25-3p and miR-451a and the downregulation of miR-320e were validated as deregulated in PanNET as compared to CP (Fig. 1 i-j-k).

### Lesion specific circulating miRNAs

**Fig. 2:**
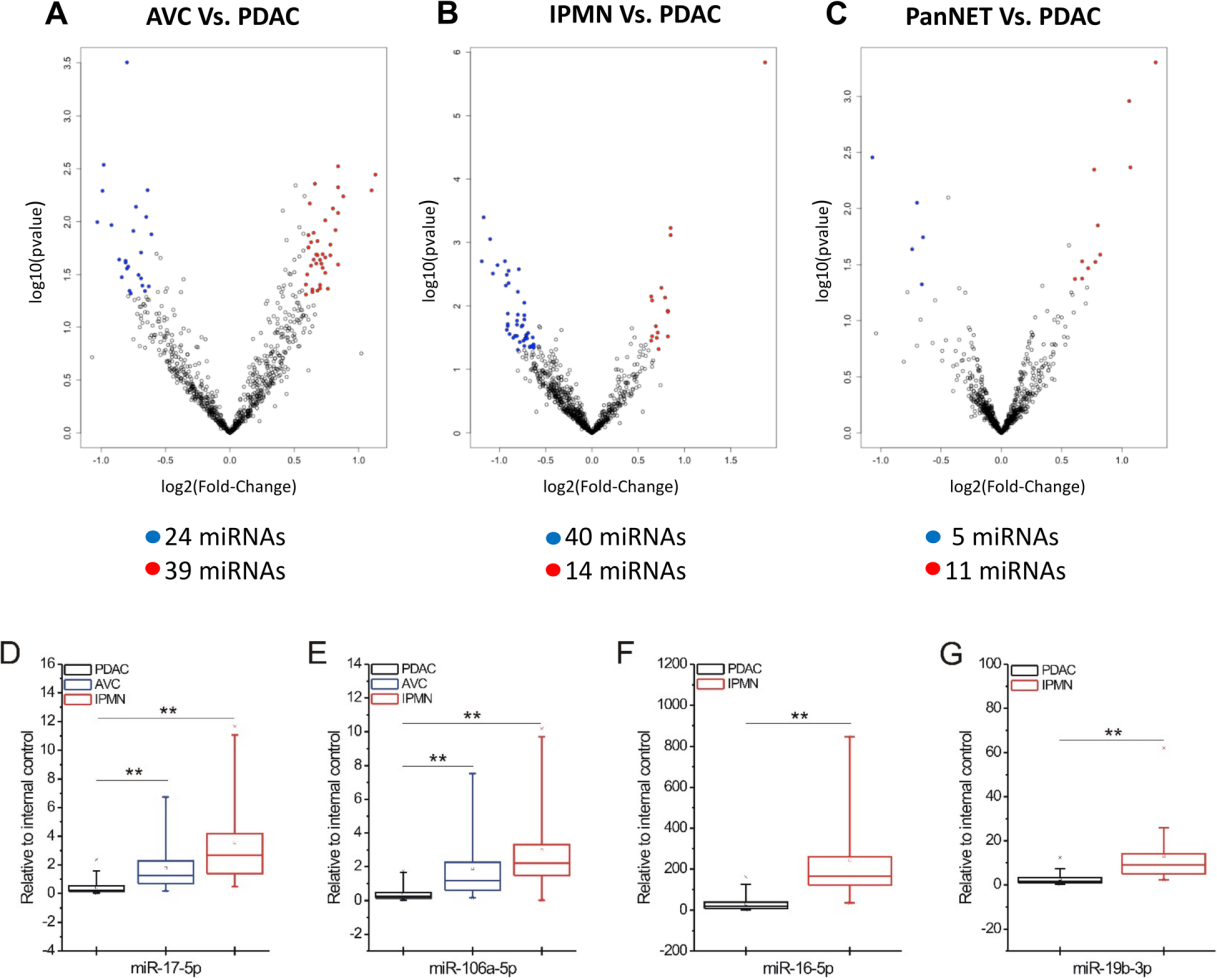
Differential expression of circulating miRNAs in pancreatic cancer patients. **a-b-c** Volcano plots of miRNA expression showing significant (*P* < 0.05), deregulated (with a |LinearFC| > 1.5) miRNAs in each comparison and an expression ≥20 counts in at least one condition. **d-e-f-g** NanoString results were validated by qRT-PCR. The differential expression of circulating miRNAs was assessed by using as a control the PDAC group with respect to the indicated pancreatic cancer groups. The square inside the box plot indicates the mean value, whereas the “x” outside the box indicates the 99-percentile. Student’s *t*-test was performed for statistical analysis. **, 0.001 < *P* ≤ 0.01

Differential miRNA profiling was also performed in order to identify relevant deregulated miRNAs between different pancreatic lesions (AVC vs PDAC, IPMN vs PDAC and PanNET vs PDAC). As reported in Fig. 2, we were able to identify 63 deregulated miRNAs between AVC and PDAC, 54 miRNAs between IPMN and PDAC and 16 miRNAs between PanNET and PDAC (Fig. 2 a-b-c). A linear fold change> 1.5 was applied as threshold for each comparison. After data filtering, we found 22 upregulated miRNAs in AVC compared to PDAC, 21 upregulated miRNAs in IPMN compared to PDAC and 7 deregulated miRNAs between PanNET and PDAC (4 upregulated and 3 downregulated miRNAs in PanNETs compared to PDAC) (Table 2 and Additional file 4). Selected miRNAs were than validated by qRT-PCR between different pancreatic lesions as reported in Additional file 3. Consistent with NanoString data, we found miR-17-5p and miR-106-5p significantly upregulated in AVC and IPMN as compared to PDAC (Fig. 2 d-e and Table 2). The upregulation of miR-16-5p and miR-19-3p in IPMN compared to PDAC was also validated as reported in Fig. 2 (f-g) and Table 2.

**Fig. 3:**
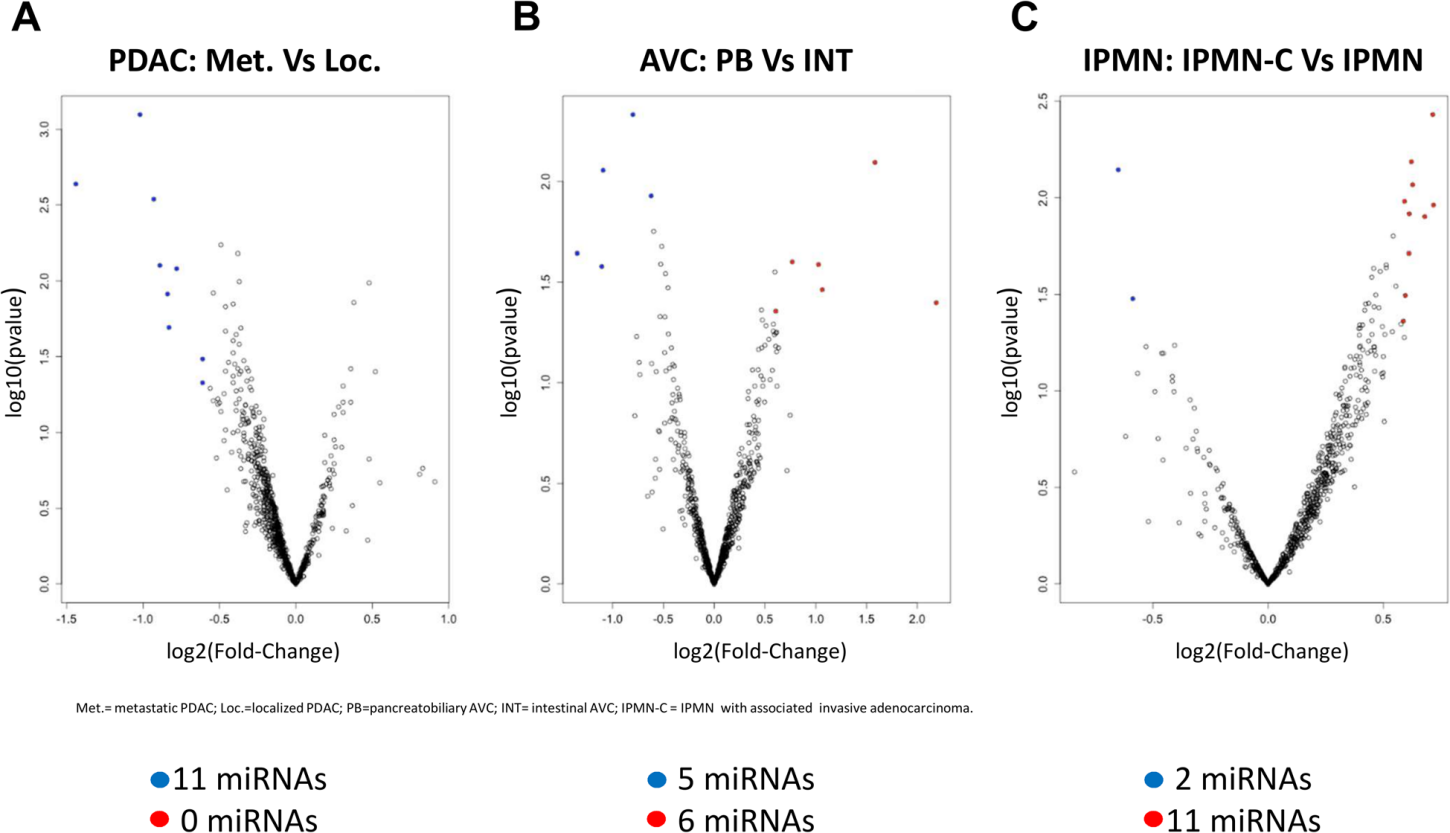
Different expression of circulating miRNAs in different biological and clinical subtypes of the same pancreatic lesion histotype. **a-b-c** Volcano plots of miRNAs expression showing significant (*P* value< 0.05), deregulated (with a |LinearFC| > 1.5) miRNAs in each comparison and an expression ≥20 counts in at least one condition

We also focused on the most deregulated circulating miRNAs associated with different biological and clinical subtypes of the same histotype: metastatic PDAC Vs localized PDAC (Met. PDAC vs Loc. PDAC), pancreatobiliary AVC vs intestinal AVC (PB AVC vs INT AVC) and IPMN with associated invasive adenocarcinoma and IPMN without associated invasive adenocarcinoma (IPMN-C VS IPMN) (Fig. 3). Using a linear fold change > 1.5 as threshold, we identified overall 9 deregulated miRNAs between metastatic and localized PDAC, 11 miRNAs between PB AVC and INT AVC and 12 miRNAs between malignant IPMN and benign IPMN (Fig. 3 a-b-c). As for previous analysis, data were filtered again leading to the identification of 9 downregulated miRNAs in metastatic PDAC compared to the localized one, 11 deregulated miRNAs between pancreatobiliary and intestinal IPMN (6 upregulated and 5 downregulated miRNAs) and 12 miRNAs between malignant and benign IPMN (10 upregulated and 2 downregulated miRNAs) (Table 2 and Additional file 4).

### In situ analysis confirmed exosomal miRNA profiling

**Fig. 4:**
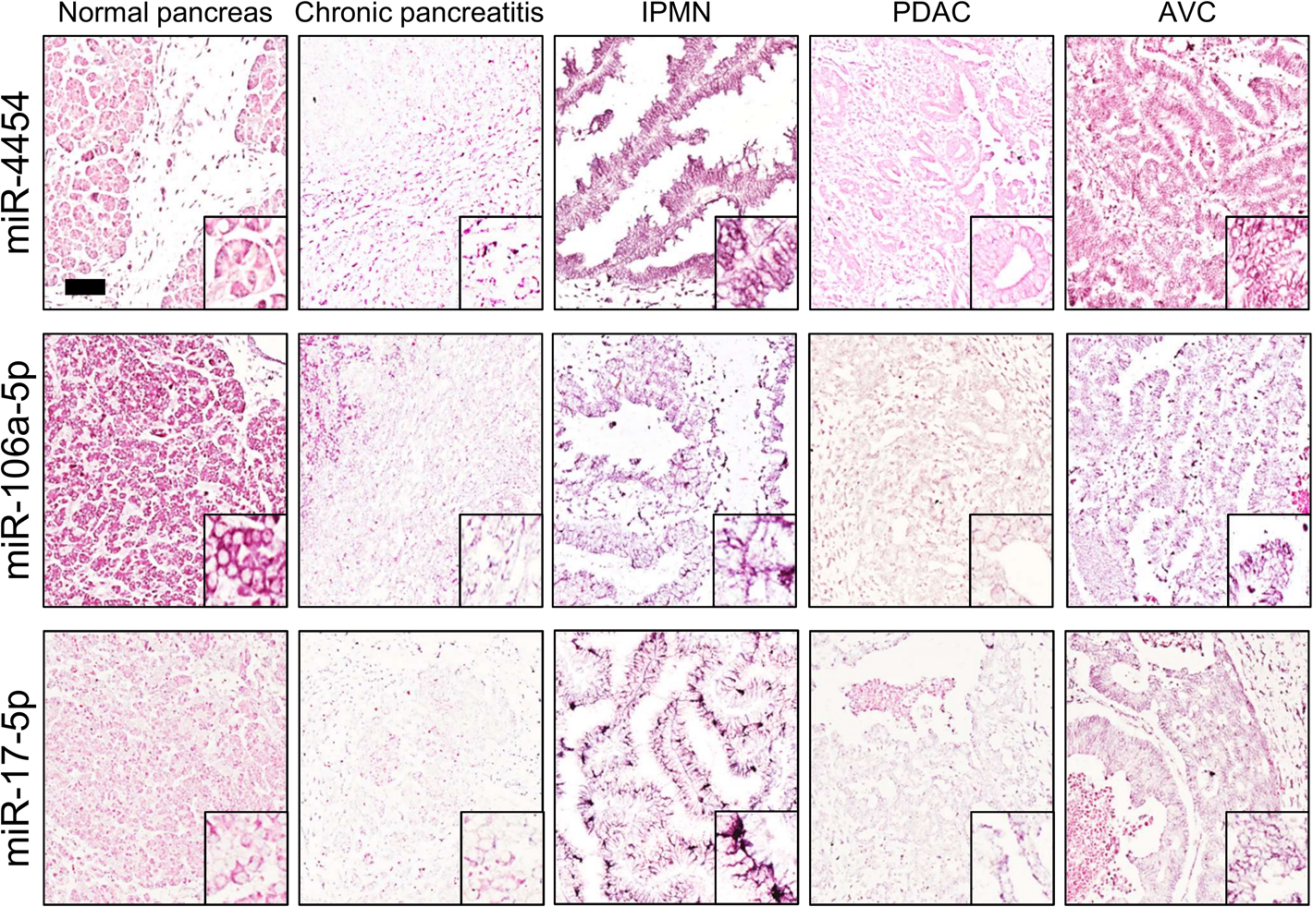
Representative in situ hybridization (ISH) of miR-4454, miR-106-5p, miR-17-5p in tissue sections of pancreatic cancers. ISH assays demonstrate a significant miRNA expression dysregulation among different tumor hystotypes. Normal grey matter specimens showed a negative/faint expression of miR-106-5p and miR-17-5p in CP. On the other hand, IPMN and AVC showed a moderate/strong expression of miR-106-5p and miR-4454. Columns denote the different tumor subtype while rows the different miRNAs analyzed. The presence of miRNA is shown by a grainy blue cytoplasmatic stain; slides counterstained in fast red. (Scale bars 200 μm; Original magnifications 10x and 5x)

To further support our findings, we performed in situ hybridization (ISH) assay on matched formalin-fixed paraffin-embedded (FFPE) tissue sections of CP, IPMN, AVC, PDAC patients and normal pancreas for miR-4454, miR-106a-5p and miR-17-5p (Fig. 4). Despite ISH analyses lack the required sensitivity to identify subtle changes in expression levels of miRNA, they largely reflected qRT-PCR results. MiR-4454, which was significantly upregulated in CP compared to PDAC through qRT-PCR, showed the same expression trend by ISH (Fig. 4). MiR-106-5p and miR-17-5p qRT-PCR data also well matched with ISH experiments. As reported in Fig. 4, miR-106-5p was more expressed in IPMN and AVC tissue section as compared to PDAC and CP, while the expression of miR-17-5p was more evident in AVC and IPMN compared to PDAC.

## Discussion

In the last few years, many studies have highlighted the involvement of miRNAs in tumorigenesis and cancer progression supporting their possible role as new biomarkers for cancers [37]. Moreover, the identification of circulating miRNAs in the body fluids such as plasma/serum underline their potential application for detection, monitoring and predicting prognosis of cancer patients [24, 38]. At present, several miRNA expression profiling studies identified miRNA signatures associated to clinico-pathological features in PDAC as staging, progression, prognosis and response to treatment both on tissues [39–43] and biofluids [44–49] while only few data have been published on miRNAs and gastroenteropancreatic neuroendocrine tumors [31, 50–52]. Genome-wide miRNA expression analyses were also performed on IPMN tissues [53–56] but only two studies evaluated blood-based miRNA expression in early stage PDAC patients versus controls [45, 55]. As for IPMN, few information has been provided on circulating miRNAs and AVC, with plasma miR-192 up-regulation in periampullary carcinoma and its correlation with tumor stage and aggressiveness as the only identified biomarker [32, 57].

Despite this huge amount of data, miRNA expression profiling has not been introduced into the clinical practice, so far, maintaining radiological imaging and biopsy as the gold standards for tumor detection and diagnosis [58]. The causes of this failure may be found in the inconsistent results observed among studies or the unfeasibility to compare each other results due to different analytic methods, detection technologies or circulating components (e. g. plasma, serum or whole blood) analyzed [53]. On this ground, we applied digital profiling of circulating exosomal miRNAs to distinct pancreatic lesions in order to establish a bioresource for biomarker development. This is one of the most extensive exosomal miRNA study able to identify simultaneously peculiar miRNA signatures associate to different pancreatic lesions and within biological and clinical different subgroup of the same tumor histotype.

Recent evidences pointed out that exosomes represent a big bioresource of miRNAs, showing several advantages comparing to cell free miRNAs. First, exosomal miRNAs are protected from degradation even if in the presence of RNase and are stable under different storage conditions [59, 60]. Secondly, it has been reported that they are representative of the matched parental tumor in terms of miRNA signatures [61, 62] and finally, it is known that malignant tissues secrete a higher amount of exosomes compared to the normal counterpart in biofluids such as plasma and urine [63, 64]. These peculiar features render exosomal miRNAs a promising candidate for miRNAs’ profile studies.

In our study, plasma-derived exosomal RNA of 140 pancreatic lesions and 15 cases of chronic pancreatitis were processed by NanoString technology. Data analysis allowed us to select differentially expressed miRNAs in the majority of comparison between histotypes and CP patients. Selected miRNAs (miR-106-5p; miR-4454; miR-7975; miR-320e; miR-19b-3p; miR-16-5p and miR-17-5p) were validated between groups by qRT-PCR underling the powerful of NanoString technique for miRNAs profiling, especially for small RNA quantities as in our case (Figs. 1 and 2). Three selected miRNAs (miR-4454; miR-106a-5p and miR-17-5p) were then validated also by in situ hybridization, which largely reflected the results of plasma profiling and showed that most of circulating miRNAs were derived from the epithelial components of the lesions.

For the first time miR-4454 was found significantly upregulated in the plasma of patients with chronic pancreatitis compared to PDAC and PanNET (Fig. 1g); notably, this result has been also validated by ISH on tissue samples, further corroborating its reliability (Fig. 4). In line with our findings, in the literature there is evidence supporting the role of miR-4454 in inflammation. For example, it has been reported in lung that miR-4454 targets include cytokines and matrix metalloproteinases that could have a relevant impact on pulmonary inflammation and fibrosis [65]. Moreover miR-4454 was also identified as mediators of facet cartilage degeneration promoting inflammatory, catabolic and cell death activity [66].

Next, we aimed to identify miRNAs associated to biological and clinical different subtypes within the same histotype. Since recent studies highlighted the role of exosomes in the formation of a premetastatic niche in the liver [67] and in tumor proliferation [68], we profiled, for the first time, exosomal miRNAs between localized and metastatic pancreatic ductal adenocarcinoma. Our analysis identified a signature of 11 miRNAs significantly downregulated in metastatic PDAC compared to localized disease (Table 2). Further functional studies should investigate the biological role of this signature as useful non-invasive tool able to monitor disease progression.

The current standard histopathologic evaluation is often not able to identify the precise origin of a periampullary lesions, including neoplasms from other subtypes as could appended for ampullary carcinomas [69]. Moreover, since AVC may be classified as pancreatobiliary or intestinal according to the histologic type of differentiation [70], we explored the possibility to identify peculiar exosomal miRNAs signatures able to discriminate between PDAC and AVC and within AVC subtypes. In particular, we found 22 exosomal miRNAs differently expressed between AVC and PDAC and 11 exosomal miRNAs between pancreatobiliary and intestinal AVC (Table 2). These signatures could represent an important bioresources for further validations aimed to discriminate between different lesions.

IPMN showed a wide histological spectrum ranging from benign adenomas to invasive carcinoma and represent an important precursor of aggressive pancreatic ductal adenocarcinoma. Since IPMN with an associated PDAC have a significantly worse prognosis compared to IPMN without PDAC and since the identification of this association is important especially prior the surgery [71], we investigated potential different exosomal miRNAs signature between these two entities identifying 13 differently expressed miRNAs between lesions (Table 2).

## Conclusion

In this study we showed that pancreatic lesions are characterized by specific exosomal miRNAs signatures. We also provided a bioresource for future explorations aimed to understand the biological and clinical relevance of such signatures.

## Supplementary information

**Additional file 1: Table S1.** Summary of clinicopathological characteristics of pancreatic neoplasm patients.

**Additional file 2: Figure S1.** Quality and size analysis of pancreatic plasma-derived exosomes.

**Additional file 3: Table S2.** Deregulated miRNAs from Nanostring profiling associated to different pancreatic lesions.

**Additional file 4: Table S3.** Deregulated miRNAs from Nanostring profiling associated to biological and clinical different subtypes within the same histotype.

## Data Availability

Supporting data and protocols are made available without restrictions from Prof. Aldo Scarpa.

## Abbreviations

PDAC: pancreatic ductal adenocarcinoma
PanNET: pancreatic neuroendocrine tumors
IPMN: intraductal papillary mucinous neoplasms
AVC: ampulla of Vater carcinomas
PanIN: pancreatic intraepithelial neoplasia
MCN: mucinous cystic neoplasm
CP: chronic pancreatitis
Met. PDAC: metastatic PDAC
Loc. PDAC: Localized PDAC
PB AVC: pancreatobiliary AVC
INT AVC: intestinal AVC
IPMN-C: IPMN with associated invasive adenocarcinoma
