# Supplementary material for: Exosomal miRNA signatures of pancreatic lesions
Source: BMC Gastroenterol. 2020 May 6;20:137. doi: 10.1186/s12876-020-01287-y (PMC7204029; doi:10.1186/s12876-020-01287-y)
Supplement: Supplementary file 2 — Additional file 2: Figure S1. Quality and size analysis of pancreatic plasma-derived exosomes. [file 12876_2020_1287_MOESM2_ESM.pdf]

A

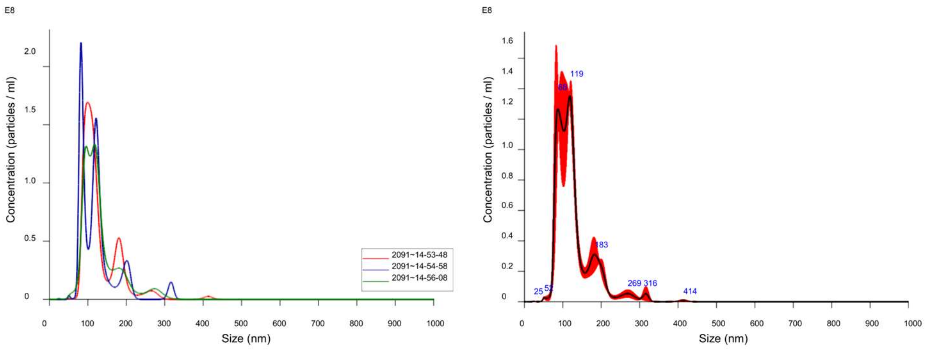

Sample	Ct RNU44	Ct RNU48
Exo human plasma	Undet.	38.2

B

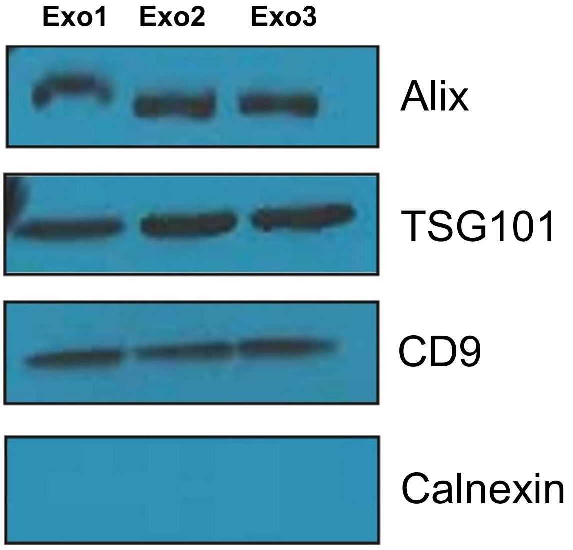

Supplementary Figure 1. Quality and size analysis of pancreatic plasma-derived exosomes. A Representative image derived from Nanosight analysis performed on pancreatic cancer patient plasma-derived exosomes, showing the quality and size of isolated vesicles. For each analyzed sample, three short videos were taken and averaged. The left panel displays the vesicle distribution for size and concentration in each individual short video (indicated by a specific color), instead the right panel shows the average result of the three videos. The numbers next to each peak indicate the average values that were determined by the analysis. The table shows that cellular miRNA normalizers RNU44 and RNU48 were not detected by qRT-PCR within isolated vesicles, indicating that our exosomal samples did not yield cell contaminants that could compromise our results. **B** Western blot results show the presence of exosomal markers Alix, TSG101 and CD9 within three representative exosomal protein lysates. The presence of the integral protein of the endoplasmic reticulum Calnexin could not be detected, confirming that our samples were not contaminated by cells..
